# Chronic Obstructive Pulmonary Disease Patients’ Acceptance in E-Health Clinical Trials

**DOI:** 10.3390/ijerph18105230

**Published:** 2021-05-14

**Authors:** Saeed M. Alghamdi, Ahmed M. Al Rajah, Yousef S. Aldabayan, Abdulelah M. Aldhahir, Jaber S. Alqahtani, Abdulaziz A. Alzahrani

**Affiliations:** 1 Department of Respiratory Care, College of Applied Health Science, Umm Al Qura University, Makkah 21955, Saudi Arabia; aaa717@student.bham.ac.uk; 2 National Heart and Lung Institute, Imperial College London, London SW3 6NP, UK; 3 Respiratory Care Department, College of Applied Medical Sciences, King Faisal University, Al-Ahsa 31982, Saudi Arabia; amalrajeh@kfu.edu.sa (A.M.A.R.); yaldabayan@kfu.edu.sa (Y.S.A.); 4 Respiratory Care Department, Faculty of Applied Medical Sciences, Jazan University, Jazan 45142, Saudi Arabia; abdulelah.aldhahir.17@ucl.ac.uk; 5 UCL Respiratory, University College London, London WC1E 6BT, UK; jaber.alqahtani.18@ucl.ac.uk; 6 Department of Respiratory Care, Prince Sultan Military College of Health Sciences, Dammam 34313, Saudi Arabia; 7 Institute of Clinical Sciences, University of Birmingham, Birmingham B15 2TT, UK

**Keywords:** systematic review, meta-analysis, telehealth, chronic obstructive pulmonary disease, COPD

## Abstract

Introduction: Telehealth (TH) interventions with Chronic Obstructive Pulmonary Disease (COPD) management were introduced in the literature more than 20 years ago with different labeling, but there was no summary for the overall acceptance and dropout rates as well as associated variables. Objective: This review aims to summarize the acceptance and dropout rates used in TH interventions and identify to what extent clinical settings, sociodemographic factors, and intervention factors might impact the overall acceptance and completion rates of TH interventions. Methods: We conducted a systematic search up to April 2021 on CINAHL, PubMed, MEDLINE (Ovid), Cochrane, Web of Sciences, and Embase to retrieve randomized and non-randomized control trials that provide TH interventions alone or accompanied with other interventions to individuals with COPD. Results: Twenty-seven studies met the inclusion criteria. Overall, the unweighted average of acceptance and dropout rates for all included studies were 80% and 19%, respectively. A meta-analysis on the pooled difference between the acceptance rates and dropout rates (weighted by the sample size) revealed a significant difference in acceptance and dropout rates among all TH interventions 51% (95% CI 49% to 52; *p* < 0.001) and 63% (95% CI 60% to 67; *p* < 0.001), respectively. Analysis revealed that acceptance and dropout rates can be impacted by trial-related, sociodemographic, and intervention-related variables. The most common reasons for dropouts were technical difficulties (33%), followed by complicated system (31%). Conclusions: Current TH COPD interventions have a pooled acceptance rate of 51%, but this is accompanied by a high dropout rate of 63%. Acceptance and dropout levels in TH clinical trials can be affected by sociodemographic and intervention-related factors. This knowledge enlightens designs for well-accepted future TH clinical trials. PROSPERO registration number CRD4201707854.

## 1. Introduction

More than 10% of the population worldwide aged 40 years or older are affected by Chronic Obstructive Pulmonary Disease (COPD) [1]. In general, COPD is caused by smoking cigarettes, which may lead to death or disability [2]. The prevalence of COPD has increased dramatically over the past 30 years and is predicted to be the third-leading cause of death by 2030 [1,2,3]. According to the Global Initiative for Chronic Obstructive Lung Disease (GOLD), COPD is a common, preventable, and treatable disease characterized by persistent airflow limitation that is usually progressive and associated with enhanced chronic inflammatory responses to noxious gases in the airways and the lungs [4]. Airflow limitations lead to worsening respiratory symptoms—exacerbation—and, often, hospitalization [5,6]. COPD patients require an appropriate management strategy that aims to minimize the frequency of hospitalizations [7].

Recently, research has focused on delivering COPD care via telehealth (TH) to offer prompt access to healthcare and to increase the capacity of COPD care [8]. Published systematic reviews have found that delivering COPD care using TH may provide a mechanism to encourage collaboration between patients and healthcare providers and may enhance patient knowledge and skills in learning how to deal with their conditions [8,9]. Moreover, it facilitates regular monitoring of patients’ clinical data, such as vital signs, to allow the healthcare team to detect any disease deterioration at an early stage before it worsens and provide the necessary care to minimize hospital admissions due to respiratory exacerbation [8,9]. Additionally, TH plays an important role in supporting public health precautions and in mitigating the spread of infections such as COVID-19 [10]. Taking the above into account, TH technologies have different applications to provide health care for COPD patients [8,11].

Using TH in COPD management has been found feasible, valuable, and accessible, but recent evidence shows variation regarding the completion of such interventions [11,12,13]. Previous clinical trials show that individuals with COPD have positive attitudes toward participating in TH interventions [14,15,16,17,18,19]. However, evidence about the impact of TH on health service outcomes or patient-related outcomes is still inconclusive [8,11]. This lack of knowledge about the effectiveness of TH for COPD management might lead to poor acceptance of TH interventions and/or a high dropout rate and withdrawal of participants from TH studies [20]. While TH use is promising in COPD management, it is unclear which factors are most associated with acceptance and dropout rates and whether these factors are trial-related, sociodemographic, or intervention-related. Therefore, our review aims to (1) assess the overall acceptance and dropout rates in TH clinical trials, (2) summarize the reasons for dropouts from TH interventions, and (3) explore factors that have an impact on overall acceptance and dropout rates.

## 2. Methods

The current systematic review was reported according to the Preferred Reporting Items for Systematic Review and Meta-analysis (PRISMA) guidelines [21].

### 2.1. The Inclusion Criteria

1. Controlled clinical trials with or without randomization that examined TH interventions;
2. Studies that include patients diagnosed with COPD (defined as forced expiratory volume in 1 s (FEV_1_)/forced vital capacity (FVC) ratio < 70%, FEV_1_ < 80% predicted);
3. The intervention included in this review is telehealth. As telehealth interventions have different labels (e.g., telemonitoring, telerehabilitation) in the literature, no restrictions have been applied on intervention labeling. TH interventions with different labels which use internet or electronic health information and communication technologies to support distance health care and/or exchange information between patients and healthcare providers were included.

### 2.2. The Exclusion Criteria

1. Studies that targeted non-COPD individuals and/or a general population;
2. Trials published in a language other than English;
3. Studies that did not describe TH, including the content of the intervention, delivery method, mode of administration, and frequency of data transmission;
4. Studies that did not report the number of COPD individuals who were approached, consented, and dropped out.

### 2.3. Search Strategy

An electronic search of the following databases up to April 2021 was undertaken to retrieve relevant articles: CINAHL, PubMed, MEDLINE (Ovid), Cochrane Library, and Embase. Medical subject headings, subject headings, and/or their combinations used in all databases were as follows: telehealth, telecare, telehomecare, telemonitoring, telerehabilitation, telemedicine, home monitoring, digital monitoring, web-based interventions, internet-based monitoring, e-health, chronic obstructive pulmonary disease, chronic obstructive lung disease, and COPD. The search was conducted in collaboration with the health sciences librarian to ensure that our search included the appropriate and necessary keywords in the review. Keywords and subject terms were customized in each database. A full search strategy from all databases is provided in Supplementary materials Files S1–S10.

### 2.4. Search Procedures

The search was performed by the main reviewer (SA), after which all articles were imported to EndNote version X9.3 (Clarivate, Philadelphia, PA, USA), and duplicates were removed. All titles and abstracts were screened by two reviewers (SA and AA). A third reviewer (YA) was available to resolve any persisting disagreements. A manual search of the reference lists of relevant studies was undertaken to identify any potentially relevant articles that were missed by the database search but that might be suitable for inclusion in the review. A full-text review of all suitable articles was undertaken, and any study that did not meet the inclusion criteria was excluded.

### 2.5. Data Extraction

A standardized Excel (Microsoft Corporation, Redmond, WA, USA) spreadsheet was created for data extraction. The spreadsheet included information on acceptance and dropout rates, as well as trial-related, sociodemographic, and intervention-related factors. Trial-related factors include study place, study design, and recruitment location. The sociodemographic factors include age, status at recruitment, and smoking history. The intervention-related factors include the components of the intervention, methods of delivery, display, frequency, and duration of the intervention. For any missing data, the authors attempted to contact the corresponding publishers of the included studies and completed the data extraction form.

The quality of the studies was defined based on the Cochrane risk-of-bias tool [22]. Data extraction and quality assessment were performed by two independent reviewers (SA and AA). Any disagreement between reviewers (SA and AA) was resolved by consensus.

### 2.6. Data Analysis

The synthesized results in this review focused on the key outcomes of interest, including the acceptance and dropout rates, reasons for dropout, and the possible factors that might impact acceptance and dropout rates. The overall acceptance rate in this paper refers to the number of participants who consented to participate divided by the number of participants who were approached to participate in the trial [23,24], and dropout rate refers to the total number of participants in each treatment arm who dropped out from the clinical trial divided by the total number of the participants who consented to participate in the clinical trial [23].

Possible explanatory trial-related factors, sociodemographic factors, and intervention-related factors that might be associated with acceptance and dropout rates were identified from the literature [25,26,27,28,29,30]. All factors were converted to binary data. Trial-related factors are categorized as the place of the study (Europe vs. non-Europe), design of the study (Randomized Control Trial vs. non-Randomized Control Trial), and the recruitment location (one recruitment location vs. more than one). The sociodemographic factors are categorized as age (<69 vs. ≥69), the status of COPD at recruitment (stable vs. non-stable), and smoking history (yes vs. no). The intervention-related factors include components of the intervention (one component vs. more than one), methods of delivery (internet-based vs. other), interactive display (interactive vs. not interactive), frequency (daily vs. weekly), and the duration of the intervention (≤20 weeks vs. >20 weeks). Overall, acceptance and dropout rates were computed for each category to compare factors. Continuous variables are expressed as mean and standard deviations. Categorical variables are expressed as frequency and percentages.

We performed an additional meta-analysis to estimate the pooled difference of acceptance and dropout rates between treatment arms. The estimation of rates weighted by the sample size in each clinical trial, and data were pooled using random-effects models. We expressed rates as proportions and 95% confidence intervals (CIs). Heterogeneity among included studies was assessed using the I-square (I^2^) value. All statistical analyses in this study were performed using STATA software (StataCorp, College Station, TX, USA). More information about the statistical methods can be found in Supplementary File S1.

## 3. Results

### 3.1. Study Selection

**Figure 1:**
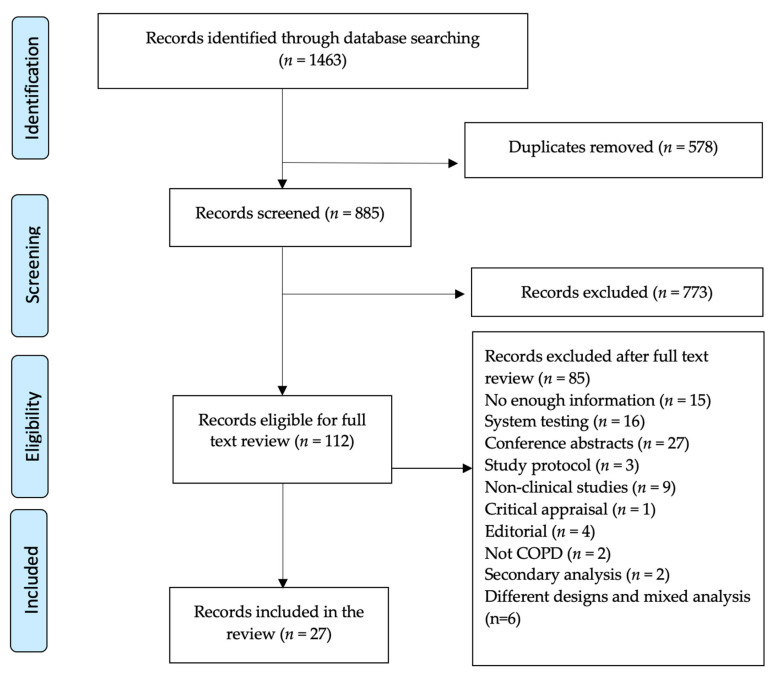
PRISMA flow diagram showing studies related to the TH (telehealth) interventions in COPD (with Chronic Obstructive Pulmonary Disease).

The search identified a total of 1463 relevant articles, 885 after duplicates had been excluded, with a total of 112 articles maintained for full-text review following the title and abstract screening. The remaining articles were read in full, and 27 articles were considered for the review, as shown in the PRISMA flow diagram (Figure 1).

### 3.2. Study Characteristics

The majority of the included studies were published in Europe (*n* = 18). Most of the studies were RCT (*n* = 24). Twenty-one out of twenty-seven clinical trials recruited patients from different clinical settings.

### 3.3. Patient Characteristics

The included trials [18,31,32,33,34,35,36,37,38,39,40,41,42,43,44,45,46,47,48,49,50,51,52,53,54,55,56] collectively comprised a total of 4157 COPD patients. Participation age (mean ± standard deviation) was 65 ± 7.1 years. In the included studies, 18 studies out of 27 (65%) reported smoking history. COPD GOLD III was the most common GOLD grade among included participants: 3118/4157 (75%). More descriptive details about the studies and sociodemographic characteristics can be found in Supplementary File S3.

### 3.4. TH Intervention Characteristics

A variety of TH interventions were provided in the included studies, either self-management programs via telemonitoring or self-management programs combined with other interventions (e.g., exercise, pulmonary rehabilitation, home care). Twenty of the studies out of twenty-seven had more than one component. Fourteen of the studies out of twenty-seven delivered interventions using web-based video or telephone calls. Eighteen of the studies out of twenty-seven delivered the intervention with daily frequencies, while nine studies out of twenty-seven delivered the intervention in weekly frequencies. The duration of the intervention ranged from three weeks to forty-eight weeks. Sixteen of the studies out of twenty-seven had an intervention of >20 weeks. The control groups, for example, were provided with the usual care, written self-management, and written exercise guidelines. More descriptive details about TH and control intervention characteristics can be found in Supplementary Files S4 and S5.

### 3.5. Acceptance and Dropout Rates

The total number of individuals with COPD approached to participate in the included clinical trial was 8085. Of these, 3928 patients were ineligible and excluded. A total of 4157 consented and enrolled in the clinical trials. Overall, the unweighted average of acceptance and dropout rates for all included studies were 80% and 19%, respectively. The acceptance and dropout rates in all included studies were stratified by factors, as provided in Table 1.

### 3.6. Meta-Analysis

An additional meta-analysis of acceptance and dropout rates in all included studies (weighted by the sample size) demonstrated significant differences in the acceptance and dropout rates between TH and control groups. As shown in Table 2, the pooled difference of the acceptance and dropout rates of TH interventions with corresponding 95% CIs was 51% (49% to 52%); *p* < 0.001 and 63% (60% to 67%); *p* < 0.001, respectively. More information about the pooled acceptance and dropout rates in each treatment arm can be found in Supplementary Files S6–S9.

### 3.7. Reasons for Dropout

After randomization or allocation to treatment, 1152 participants completed their TH interventions, and 946 withdrew before the end of the study. Of these, only 513 participants provided the reasons for study withdrawal. Reasons for dropout classified as TH-related reasons and individual-related reasons are shown in Table 3.

### 3.8. Quality Assessment

Quality assessment was performed using the Cochrane risk-of-bias assessment tool [22]. The studies included showed variation in their risk of bias, but most were limited by a lack of blinding. A summary of our judgments on the potential risk of bias can be found in Supplementary File S10.

## 4. Discussion

In the context of COPD, exploring the current acceptance and dropout rates of a different form of TH intervention and its associated factors is a crucial objective in terms of whether TH intervention is feasible or not; furthermore, these data have not been previously pooled in forest plots to summarize the overall acceptance and dropout rates. Our findings revealed that the overall average of acceptance and dropout rates was reasonable for patients with COPD, considering the trial-related, sociodemographic, and intervention-related factors. The findings indicated that 20% of the eligible participants were expected to refuse the TH interventions, and once participants were allocated to TH interventions, around 19% were expected to drop out and not complete the clinical trial. The weighted acceptance rate was 51% in TH groups, suggesting that TH interventions were accepted among people with COPD. Moreover, the dropout rate was higher in the TH intervention groups versus control groups (63% vs. 37%). This finding was expected because most of the dropout reasons from TH studies were TH intervention-related reasons. However, we identified certain factors that have the potential to improve acceptance and reduce the dropout rate of TH interventions with COPD.

In the context of clinical research on COPD, the acceptance rate was higher in multicenter short-term (<20 weeks) trials that were done in European countries. A possible explanation is that advanced countries have good adoption and success rates of TH implementations compared to low- and middle-class countries [57,58,59], but it could also be due to other financial, organizational, and economic factors since large multicenter clinical trials need considerable research funding, collaboration between clinical settings, qualified researchers, and a large number of patients [57,60].

Moreover, a high acceptance rate in the studies that remotely monitored people with COPD after hospital discharge has been observed. This is due to the benefits of TH in detecting COPD deterioration in early stages and minimizing hospital readmission due to exacerbations. Moreover, some evidence found that offering TH interventions to people with COPD seems to be an opportunity to provide quality healthcare and to minimize unnecessary hospitalization [61,62]. A patient’s smoking history was identified as an additional explanatory factor to increase TH acceptability. Similar findings were found by Watson et al. that recruiting smokers in TH interventions for less than 20 weeks significantly increased the acceptance rate [63]. This result could be because these patients have been chronic smokers for years, and smoking cessation interventions are not working well for them. The increased participation of smokers with COPD in TH interventions could indicate that those patients are searching for more accessible smoking cessation interventions [64].

Furthermore, the high acceptance rate of TH that provides several components (e.g., telemonitoring, tele-coaching, and self-management education) has been inspected. A possible explanation is that these TH interventions provide more individual-based interventions that meet the patient’s needs. There were similar findings in the literature about using more approaches to address the patient’s needs (e.g., uptake pulmonary rehabilitation), resulting in greater patient engagement and satisfaction [65]. Moreover, TH interventions that provide daily monitoring through convenient methods (e.g., telephone calls and text messages) had a high acceptance rate because they used a simple method of communication [32].

Refusal to complete TH interventions is primarily attributed to the interventions themselves [66,67]. It was noted that TH interventions with multiple components were fraught with complexities and technical difficulties that have resulted in decreased treatment sessions or even termination [18,32]. This could lead to participant dissatisfaction and, ultimately, dropping out of the study. Essential steps at the designing and implementation levels are needed to plan and enable more technical support during the delivery of TH interventions [68]. Another possible explanation for dropout rates could be the discrepancy between the patients’ expectations and their abilities to operate and use TH [69]. Most of the studies did not provide training sessions for the participants; specifically, there were no measurements of participants’ competency in using the technology or cognitive abilities to use TH. Thus, attention must be paid to accommodating these factors in the design of future TH interventions [70].

Telehealth application is important, and COPD management guidelines have supported its implementation [71,72,73]. Current systematic reviews provide a comprehensive description and summary of the acceptance and dropout rates of different forms of TH interventions for patients with COPD. Generally, TH interventions are acceptable among the COPD population [11,20,23,24,67,68,74,75]. Nevertheless, methodological queries remain regarding the design of a more acceptable and feasible TH intervention, the best strategy to provide TH interventions (individual vs. community), components of TH interventions (solo vs. joint other treatments), technological aspects (classic vs. advanced), and COPD phenotypes (stable vs. non-stable) that will obtain more benefits from TH interventions. This suggests that we need a more in-depth understanding of the acceptability and feasibility of TH interventions developed for COPD patients, as well as an in-depth understanding of the sociodemographic characteristics of the COPD population and their cognitive abilities when we propose TH solutions [75]. Here, the high dropout rates in TH interventions happened due to both intervention-related reasons and patient-related reasons. An additional contribution of this review is to inform future clinical trials regarding the acceptance and dropout rates of existing TH trials in the context of COPD. Moreover, this review will help researchers to identify the reasons that prevent individuals with COPD from completing TH interventions, and it provides evidence to guide future researchers in designing prospective TH clinical trials accordingly.

## 5. Limitations

Some limitations must be considered when attempting to interpret the results of the current review. There was a lack of information and incomplete data across all included studies, which impeded the possibility of exploring more variables that might influence or be influenced by the acceptance and dropout rate. Moreover, there was considerable variation in the quality of the included studies, which could limit the ability to generalize the results of this review.

## 6. Conclusions

Current TH interventions with COPD have a pooled acceptance rate of 51%, but this is accompanied by a high dropout rate of 63%. Acceptance and dropout levels in TH clinical trials may be affected by sociodemographic and intervention-related factors. Taken together, continued efforts are needed to improve patients’ acceptance by understanding and mitigating the issues contributing to the acceptance and dropout rates that would support the preparation and establishment of effective and well-accepted TH interventions.

## Figures and Tables

**Table 1:** Overall acceptance and dropout rates across included studies stratified by trial-related, sociodemographic, and intervention-related factors (*n* = 27) ^a^.

Factors	Number of Studies (%)	TH Acceptance Rate (Mean ± SD)	TH Dropout Rate (Mean ± SD)
**Trial-related factors**			
**Place of the study**			
Europe	18 (65%)	82% ± 14%	19% ± 14%
Non-Europe	9 (35%)	76% ± 14%	19% ± 16%
**Study design**			
Randomized clinical trial	24 (88%)	81% ± 14%	18% ± 14%
Non-Randomized clinical trial	3 (12%)	77% ± 18%	27% ± 14%
**Recruitment location**			
One location	21 (78%)	79% ± 14%	20% ± 14%
More than one	6 (22%)	83% ± 19%	16% ± 19%
**Sociodemographic factors**			
**COPD status at recruitment**			
Stable	18 (65%)	80% ± 16%	19% ± 16%
Non-stable	9 (35%)	81% ± 12%	18% ± 12%
**Smoking history**			
Yes	18 (65%)	82% ±14%	17% ± 14%
No	9 (35%)	76% ±16%	23% ± 15%
**Age**			
<69	5 (18%)	83% ± 10%	16% ± 10%
≥69	22(81%)	80% ± 15%	20% ± 15%
**Intervention-related factors**			
**Telehealth component (s)**			
One component	7 (25%)	78% ± 18%	21% ± 18%
More than one	20 (75%)	81% ± 13%	18% ± 13%
**Methods of delivery**			
Web-based	14 (51%)	78% ± 17%	21% ± 17%
Other	13 (49%)	82% ± 13%	17% ± 13%
**Interactive display**			
Interactive	14 (51%)	78% ± 17%	21% ± 17%
Not interactive	13 (49%)	83% ± 11%	16% ± 11%
**Frequency of TH**			
Daily	18 (65%)	81% ± 16%	18% ± 16%
Weekly	9 (35%)	78% ± 12%	21% ± 12%
**Duration of TH**			
20 weeks or less	11 (40%)	86% ± 12%	14% ± 12%
More than 20 weeks	16 (60%)	77% ± 12%	23% ± 12%

^a^ Data presented as frequency and percentages or means and standard deviations.

**Table 2:** Overall weighted acceptance and dropout rates of all included studies (*n* = 27) ^a^.

Overall Rates	Weighted (Estimation)	S.E.	*p*-Value	95% CIs
Acceptance rate in TH	51%	0.2	<0.001	49% to 52%
Acceptance rate in control	49%	0.3	<0.001	48% to 51%
Dropout rate in TH	63%	0.2	<0.001	60% to 67%
Dropout rate in control	37%	0.3	<0.001	33% to 40%

^a^ S.E: standard error.

**Table 3:** Most common reasons for dropout from TH interventions (*n* = 513) ^a^.

Dropout Reasons	Number of Patients (%)
**TH-related reasons**	
Technical difficulties	122 (24%)
Complicated system	117 (23%)
Time constraints	9 (2%)
**Individual-related reasons**	
Hospital admission	138 (27%)
Deceased	68 (13%)
Not interested in continuing	45 (9%)
Moved from the study location	14 (3%)

^a^ Data presented as frequency and percentage.

## Data Availability

Not applicable.

## Supplementary Materials

The following are available online at https://www.mdpi.com/article/10.3390/ijerph18105230/s1, File S1: Search Strategies of Databases, File S2: More Information about Statistical Methods for Acceptance and Dropout Rates, File S3: Study and Sociodemographic-Related Characteristics among Included Studies, File S4: A Detailed Description of TH Interventions and Parallel Groups, File S5: TH Intervention Characteristics of Included Studies, File S6: Forest Plot of Acceptance Rates in TH Interventions, File S7: Forest Plot of Dropout Rates in TH Interventions, File S8: Forest Plot of Acceptance Rates in Controls, File S9: Forest Plot of Dropout Rates in Controls, File S10: Assessment Risk of Bias for Included Studies.

## Abbreviations

TH	Telehealth
TM	Telemonitoring
SF	Self-management
COPD	Chronic Obstructive Pulmonary Disease
RCT	Randomized control trials
NRCT	Non-randomized control trials
