# Supplementary material for: Chronic Obstructive Pulmonary Disease Patients’ Acceptance in E-Health Clinical Trials
Source: Int J Environ Res Public Health. 2021 May 14;18(10):5230. doi: 10.3390/ijerph18105230 (PMC8156037; doi:10.3390/ijerph18105230)
Supplement: Supplementary file 1 [file ijerph-18-05230-s001.zip › Supplementary File S1.pdf]

Multimedia Appendix 1. Search strategies of databases

Ovid MEDLINE - up to January 2021

1.	Pulmonary Disease, Chronic Obstructive/ or Lung diseases, obstructive/ or Obstructive airway disease/ or Chronic obstructive lung disease/
2.	Emphysema/ or Pulmonary Emphysema/ or Bronchitis, Chronic/ or Bronchitis/
3.	Chronic Obstructive Pulmonary* Disease*.mp.
4.	Chronic Obstructive lung* disease*.mp.
5.	(COPD or COAD or chronic bronchi* or emphysema* or hyperlucent lung*).mp.
6.	or/1-5
7.	telehealth* tele-health*.mp.
8.	telecare* tele-care*.mp.
9.	telemonitoring* tele-monitoring*.mp.
10.	telerehabilitation* tele-rehabilitation*.mp.
11.	telemedicine* tele-medicine*mp.
12.	home monitoring*.mp.
13.	digital monitoring*.mp.
14.	web-based intervention*.mp.
15.	internet-based monitoring*.mp.
16.	ehealth or Ehealth or e-health*.mp.
17.	or/7-16
18.	6 and 17
19.	limit 18 to (english language and humans)

EMBASE – up to January 2021

1.	Pulmonary Disease, Chronic Obstructive/ or Lung diseases, obstructive/ or Obstructive airway disease/ or Chronic obstructive lung disease/
2.	Emphysema/ or Pulmonary Emphysema/ or Bronchitis, Chronic/ or Bronchitis/
3.	Chronic Obstructive Pulmonary* Disease*.mp.
4.	Chronic Obstructive lung* disease*.mp.
5.	(COPD or COAD or chronic bronchi* or emphysema* or hyperlucent lung*).mp.
6.	or/1-5
7.	telehealth* tele-health*.mp.
8.	telecare* tele-care*.mp.

9.	telemonitoring* tele-monitoring*.mp.
10.	telerehabilitation* tele-rehabilitation*.mp.
11.	telemedicine* tele-medicine*mp.
12.	home monitoring*.mp.
13.	digital monitoring*.mp.
14.	web-based intervention*.mp.
15.	internet-based monitoring*.mp.
16.	ehealth or Ehealth or e-health*.mp.
17.	or/7-16
18.	6 and 17
19.	limit 18 to (english language and humans)

CINAHL (EBSCO) - up to January 2021

1. (MM "Lung Diseases, Obstructive") OR (MM "Pulmonary Disease, Chronic Obstructive")
2. (MM "Emphysema") OR (MM "Bronchitis, Chronic") OR (MM "Bronchitis")
3. S1 OR S2
4. (TX "telehealth*") OR (MM" tele-health")
5. (TM" telecare") OR (MM" tele-care")
6. (TX" telehomecare*") OR (MM" tele-homecare")
7. (TX" telerehabilitation") OR (MM" tele-rehabilitation")
8. (TM" telemedicine*") OR (MM" tele-medicine")
9. (TX" home monitoring*")
10. (TX" digital monitoring*")
11. (TX" web-based interventions*") OR (MM" internet-based interventions")
12. (TX" Ehealth*") OR (MM" e-health*") OR (MM" ehealth*")
13. S4 OR S5 OR S6 OR S7 OR S8 OR S9 OR S10 OR S11 OR S11 OR S12
14. S3 AND S13
15. Limit S14 to Language: English

Cochrane Database of Systematic Reviews – up to January 2021

ID	Search
#1	(telehealth):ti,ab,kw (Word variations have been searched)
#2	telecare
#3	telehomecare
#4	telerehabilitation
#5	telemedicine
#6	home monitoring
#7	digital monitoring
#8	web-based intervention
#9	internet-based monitoring
#10	E-health
#11	#1 or #2 or #3 or #4 or #5 or #6 or #7 or #8 or #9 or #10
#12	Chronic Obstructive Pulmonary Disease
#13	Chronic Obstructive lung disease
#14	COPD
#15	#12 or #13 or #14
#16	#15 and #16

PubMed – up to January 2021

1. "telemedicine"[MeSH Terms] OR "telemedicine"[All Fields] OR "telehealth"[All Fields]
2. telecare[All Fields]
3. telehomecare[All Fields]
4. "telerehabilitation"[MeSH Terms] OR "telerehabilitation"[All Fields]
5. "telemedicine"[MeSH Terms] OR "telemedicine"[All Fields]
6. home[All Fields] AND monitoring[All Fields]

7. digital[All Fields] AND monitoring[All Fields]
8. "internet-based intervention"[MeSH Terms] OR ("internet-based"[All Fields] AND "intervention"[All Fields]) OR "internet-based intervention"[All Fields] OR ("web"[All Fields] AND "based"[All Fields] AND "intervention"[All Fields]) OR "web based intervention"[All Fields]
9. E-health [All Fields]
10. "pulmonary disease, chronic obstructive"[MeSH Terms] OR ("pulmonary"[All Fields] AND "disease"[All Fields] AND "chronic"[All Fields] AND "obstructive"[All Fields]) OR "chronic obstructive pulmonary disease"[All Fields] OR ("chronic"[All Fields] AND "obstructive"[All Fields] AND "pulmonary"[All Fields] AND "disease"[All Fields])
11. "pulmonary disease, chronic obstructive"[MeSH Terms] OR ("pulmonary"[All Fields] AND "disease"[All Fields] AND "chronic"[All Fields] AND "obstructive"[All Fields]) OR "chronic obstructive pulmonary disease"[All Fields] OR ("chronic"[All Fields] AND "obstructive"[All Fields] AND "lung"[All Fields] AND "disease"[All Fields]) OR "chronic obstructive lung disease"[All Fields]
12. "pulmonary disease, chronic obstructive"[MeSH Terms] OR ("pulmonary"[All Fields] AND "disease"[All Fields] AND "chronic"[All Fields] AND "obstructive"[All Fields]) OR "chronic obstructive pulmonary disease"[All Fields] OR "copd"[All Fields]

Web of sciences – up to January 2021

1. TOPIC: (telehealth)

Indexes=SCI-EXPANDED, SSCI, A&HCI, CPCI-S, CPCI-SSH, ESCI Timespan=All years

2. TOPIC: (telecare)

Indexes=SCI-EXPANDED, SSCI, A&HCI, CPCI-S, CPCI-SSH, ESCI Timespan=All years

3. TOPIC: (telehomecare)

Indexes=SCI-EXPANDED, SSCI, A&HCI, CPCI-S, CPCI-SSH, ESCI Timespan=All years

4. TOPIC: (telerehabilitation)

Indexes=SCI-EXPANDED, SSCI, A&HCI, CPCI-S, CPCI-SSH, ESCI Timespan=All years

5. TOPIC: (telemedicine)

Indexes=SCI-EXPANDED, SSCI, A&HCI, CPCI-S, CPCI-SSH, ESCI Timespan=All years

6. TOPIC: (home monitoring)

Indexes=SCI-EXPANDED, SSCI, A&HCI, CPCI-S, CPCI-SSH, ESCI Timespan=All years

7. TOPIC: (digital monitoring)

Indexes=SCI-EXPANDED, SSCI, A&HCI, CPCI-S, CPCI-SSH, ESCI Timespan=All years

8. TOPIC: (web-based intervention)

Indexes=SCI-EXPANDED, SSCI, A&HCI, CPCI-S, CPCI-SSH, ESCI Timespan=All years

9. TOPIC: (internet-based monitoring)

Indexes=SCI-EXPANDED, SSCI, A&HCI, CPCI-S, CPCI-SSH, ESCI Timespan=All years

10. TOPIC: (E-health)

Indexes=SCI-EXPANDED, SSCI, A&HCI, CPCI-S, CPCI-SSH, ESCI Timespan=All years

11. #10 OR #9 OR #8 OR #7 OR #6 OR #5 OR #4 OR #3 OR #2 OR #1
Indexes=SCI-EXPANDED, SSCI, A&HCI, CPCI-S, CPCI-SSH, ESCI Timespan=All years

12. TOPIC: (Chronic Obstructive Pulmonary Disease)
Indexes=SCI-EXPANDED, SSCI, A&HCI, CPCI-S, CPCI-SSH, ESCI Timespan=All years

13. TOPIC: (Chronic Obstructive lung disease)
Indexes=SCI-EXPANDED, SSCI, A&HCI, CPCI-S, CPCI-SSH, ESCI Timespan=All years

14. TOPIC: (COPD)
Indexes=SCI-EXPANDED, SSCI, A&HCI, CPCI-S, CPCI-SSH, ESCI Timespan=All years

15. #14 OR #13 OR #12
Indexes=SCI-EXPANDED, SSCI, A&HCI, CPCI-S, CPCI-SSH, ESCI Timespan=All years

16. #15 AND #11
Indexes=SCI-EXPANDED, SSCI, A&HCI, CPCI-S, CPCI-SSH, ESCI Timespan=All years
