# Supplementary material for: Chronic Obstructive Pulmonary Disease Patients’ Acceptance in E-Health Clinical Trials
Source: Int J Environ Res Public Health. 2021 May 14;18(10):5230. doi: 10.3390/ijerph18105230 (PMC8156037; doi:10.3390/ijerph18105230)

Multimedia Appendix 2. More information about statistical methods for acceptance and dropout rates.

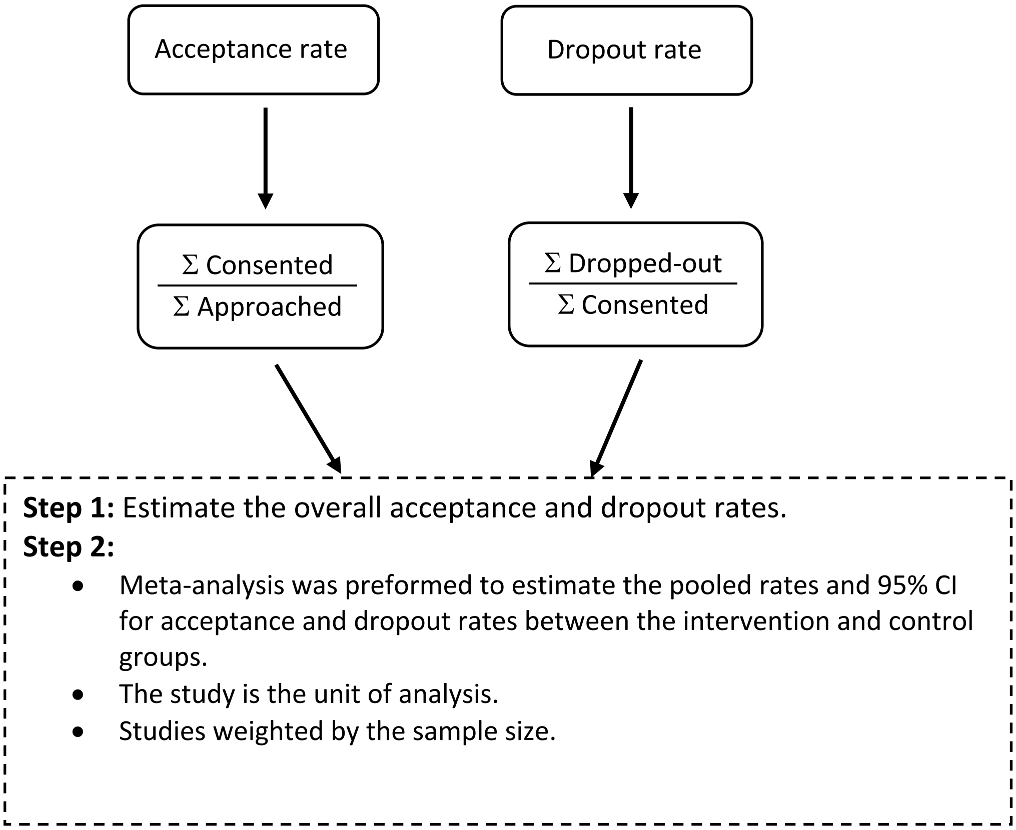

Supplement: Supplementary file 1 [file ijerph-18-05230-s001.zip › Supplementary File S2.pdf]
