# Supplementary material for: Chronic Obstructive Pulmonary Disease Patients’ Acceptance in E-Health Clinical Trials
Source: Int J Environ Res Public Health. 2021 May 14;18(10):5230. doi: 10.3390/ijerph18105230 (PMC8156037; doi:10.3390/ijerph18105230)
Supplement: Supplementary file 1 [file ijerph-18-05230-s001.zip › Supplementary File S3.pdf]

Multimedia appendix 3. Study and sociodemographic related characteristics among included studies

Authors, year	Country	Design	Duration	Total N	Mean age (SD)	Status	GOLD grade (Severity of COPD FEV1%)			
							I	II	III	IV
Antoniades et al., 2012[1]	Australia	RCT	12 months	44	70 (9)	Stable	0	44	0	0
Berkhof et al., 2015 [2]	Netherland	RCT	6 months	101	68 (9)	Stable/nonstable	0	0	101	0
Calvo et al, 2014 [3]	Spain	RCT	7 months	60	75 (9)	Stable	0	0	0	60
Cameron-Tucker et al.,2016 [4]	Australia	RCT	2 months	65	69 (8)	Stable/nonstable	4	22	24	8
Chau et al., 2012[5]	Hong Kong	RCT	2 months	40	72 (9)	Stable	0	10	13	17
De San Miguel et al, 2013 [6]	Australia	RCT	6 months	80	71(7)	Stable	0	0	0	0
Dinesen et al 2012 [7]	Denmark	RCT	4 months	111	68 (9)	Stable	0	0	111	0
Farmer et al., 2017 [8]	UK	RCT	12 months	166	69(7)	Stable	0	0	166	0
Halpin et al., 2011[9]	UK	RCT	4 months	79	69(6)	Stable	0	39	40	0
Ho et al., 2016 [10]	Taiwan	RCT	2 months	106	80(7)	Stable/nonstable	0	69	37	0
Jakobsen et al., 2015 [11]	Denmark	RCT	6 months	57	70(8)	Nonstable	0	0	0	57
Koff et al., 2009 [12]	USA	RCT	3 months	40	66 (9)	Stable	0	0	20	420
Lewis et al., 2011[13]	UK	RCT	6 months	40	67 (9)	Stable	0	0	40	0
Lilholt et al., 2017 [14]	Denmark	RCT	12 months	1225	69 (9)	Stable	0	0	1225	0
McDowell et al ,2015 [15]	UK-Ireland	RCT	6 months	110	69 (7)	Stable	0	0	100	0
Nield et al., 2012 [16]	UK-Ireland	RCT	1 month	22	64 (8)	Stable	0	22	0	0
Pedone et al., 2013 [17]	Italy	RCT	9 months	99	74 (6)	Stable	0	99	0	0
Pinnock et al., 2013 [18]	UK-Scotland	RCT	12 months	256	69 (8)	Stable/nonstable	0	88	87	81
Ringbeak et al., 2015 [19]	Denmark	RCT	6 months	281	69 (9)	Nonstable	0	0	280	0
Ringbeak etal.,2016 [20]	Denmark	NRCT	7-10 week	115	68 (9)	Stable	0	0	115	0
Schou et al., 2013 [21]	Denmark	RCT	3 months	44	68 (1)	Nonstable	0	0	44	0
Shany et al ,2017 [22]	Australia	RCT	12 months	42	72 (7)	Stable/nonstable	0	0	24	0
Stickland et al., 2011 [23]	Canada	RCT	2 months	409	69 (8)	Stable	30	138	150	91
Tabak et al., 2014 [24]	Netherland	RCT	9 months	29	64 (9)	Nonstable	0	0	29	0
Trappenburg et al.,2008 [25]	Netherland	NRCT	6 months	165	69 (8)	Stable	0	0	165	0

Tsai et al.,2017 [26]	Australia	RCT	6 months	37	73 (8)	Stable	11	12	13	0
Vianello et al., 2016 [27]	Italy	RCT	12 months	334	76(6)	Nonstable	0	0	334	0

Footnotes: N: Number; RCT: Randomised clinical trials; NRCT: Non-randomised clinical trial.

References:

1. Antoniades NC, Rochford PD, Pretto JJ, Pierce RJ, Gogler J, Steinkrug J, et al. Pilot study of remote telemonitoring in COPD. TELEMEDICINE and e-HEALTH. 2012;18(8):634-40.
2. Berkhof FF, van den Berg JW, Uil SM, Kerstjens HA. Telemedicine, the effect of nurse-initiated telephone follow up, on health status and health-care utilization in COPD patients: A randomized trial. Respirology. 2015;20(2):279-85.
3. Calvo GS, Gómez-Suárez C, Soriano J, Zamora E, Góntalez-Gamarra A, González-Béjar M, et al. A home telehealth program for patients with severe COPD: the PROMETE study. Respiratory medicine. 2014;108(3):453-62.
4. Cameron-Tucker HL, Wood-Baker R, Joseph L, Walters JA, Schüz N, Walters EH. A randomized controlled trial of telephone-mentoring with home-based walking preceding rehabilitation in COPD. International journal of chronic obstructive pulmonary disease. 2016;11:1991.
5. Chau JP-C, Lee DT-F, Yu DS-F, Chow AY-M, Yu W-C, Chair S-Y, et al. A feasibility study to investigate the acceptability and potential effectiveness of a telecare service for older people with chronic obstructive pulmonary disease. International journal of medical informatics. 2012;81(10):674-82.
6. De San Miguel K, Smith J, Lewin G. Telehealth remote monitoring for community-dwelling older adults with chronic obstructive pulmonary disease. Telemedicine and e-Health. 2013;19(9):652-7.
7. Dinesen B, Haesum LK, Soerensen N, Nielsen C, Grann O, Hejlesen O, et al. Using preventive home monitoring to reduce hospital admission rates and reduce costs: a case study of telehealth among chronic obstructive pulmonary disease patients. Journal of telemedicine and telecare. 2012;18(4):221-5.
8. Farmer A, Williams V, Velardo C, Shah SA, Yu L-M, Rutter H, et al. Self-management support using a digital health system compared with usual care for chronic obstructive pulmonary disease: randomized controlled trial. Journal of medical Internet research. 2017;19(5):e144.
9. Halpin DM, Laing-Morton T, Spedding S, Levy ML, Coyle P, Lewis J, et al. A randomised controlled trial of the effect of automated interactive calling combined with a health risk forecast on frequency and severity of exacerbations of COPD assessed clinically and using EXACT PRO. Primary Care Respiratory Journal. 2011;20(3):324-31.
10. Ho T-W, Huang C-T, Chiu H-C, Ruan S-Y, Tsai Y-J, Yu C-J, et al. Effectiveness of telemonitoring in patients with chronic obstructive pulmonary disease in Taiwan-A randomized controlled trial. Scientific reports. 2016;6:23797.
11. Jakobsen AS, Laursen LC, Rydahl-Hansen S, Østergaard B, Gerds TA, Emme C, et al. Home-based telehealth hospitalization for exacerbation of chronic obstructive pulmonary disease: findings from “the virtual hospital” trial. Telemedicine and E-health. 2015;21(5):364-73.
12. Koff P, Jones RH, Cashman JM, Voelkel NF, Vandivier R. Proactive integrated care improves quality of life in patients with COPD. European Respiratory Journal. 2009;33(5):1031-8.
13. Lewis KE, Annandale JA, Warm DL, Rees SE, Hurlin C, Blyth H, et al. Does home Telemonitoring after pulmonary rehabilitation reduce healthcare use in optimized COPD?? A pilot randomized trial. COPD: Journal of Chronic Obstructive Pulmonary Disease. 2011;7(1):44-50.
14. Lilholt PH, Udsen FW, Ehlers L, Hejlesen OK. Telehealthcare for patients suffering from chronic obstructive pulmonary disease: effects on health-related quality of life: results from the Danish ‘TeleCare North’cluster-randomised trial. BMJ open. 2017;7(5):e014587.
15. McDowell JE, McClean S, FitzGibbon F, Tate S. A randomised clinical trial of the effectiveness of home-based health care with telemonitoring in patients with COPD. Journal of telemedicine and telecare. 2015;21(2):80-7.
16. Nield M, Hoo GWS. Real-time telehealth for COPD self-management using Skype™. COPD: Journal of Chronic Obstructive Pulmonary Disease. 2012;9(6):611-9.

17. Pedone C, Chiurco D, Scarlata S, Incalzi RA. Efficacy of multiparametric telemonitoring on respiratory outcomes in elderly people with COPD: a randomized controlled trial. BMC health services research. 2013;13(1):82.
18. Pinnock H, Hanley J, McCloughan L, Todd A, Krishan A, Lewis S, et al. Effectiveness of telemonitoring integrated into existing clinical services on hospital admission for exacerbation of chronic obstructive pulmonary disease: researcher blind, multicentre, randomised controlled trial. Bmj. 2013;347:f6070.
19. Ringbæk T, Green A, Laursen LC, Frausing E, Brøndum E, Ulrik CS. Effect of tele health care on exacerbations and hospital admissions in patients with chronic obstructive pulmonary disease: a randomized clinical trial. International journal of chronic obstructive pulmonary disease. 2015;10:1801.
20. Ringbaek TJ, Lavesen M, Lange P. Tablet computers to support outpatient pulmonary rehabilitation in patients with COPD. European clinical respiratory journal. 2016;3(1):31016.
21. Schou L, Østergaard B, Rydahl-Hansen S, Rasmussen LS, Emme C, Jakobsen AS, et al. A randomised trial of telemedicine-based treatment versus conventional hospitalisation in patients with severe COPD and exacerbation–effect on self-reported outcome. Journal of telemedicine and telecare. 2013;19(3):160-5.
22. Shany T, Hession M, Pryce D, Roberts M, Basilakis J, Redmond S, et al. A small-scale randomised controlled trial of home telemonitoring in patients with severe chronic obstructive pulmonary disease. Journal of telemedicine and telecare. 2017;23(7):650-6.
23. Stickland MK, Jourdain T, Wong EY, Rodgers WM, Jendzjowsky NG, MacDonald GF. Using Telehealth technology to deliver pulmonary rehabilitation to patients with chronic obstructive pulmonary disease. Canadian respiratory journal. 2011;18(4):216-20.
24. Tabak M, Brusse-Keizer M, van der Valk P, Hermens H, Vollenbroek-Hutten M. A telehealth program for self-management of COPD exacerbations and promotion of an active lifestyle: a pilot randomized controlled trial. International journal of chronic obstructive pulmonary disease. 2014;9:935.
25. Trappenburg JC, Niesink A, de Weert-van Oene GH, van der Zeijden H, van Snippenburg R, Peters A, et al. Effects of telemonitoring in patients with chronic obstructive pulmonary disease. Telemedicine and e-Health. 2008;14(2):138-46.
26. Tsai LLY, McNamara RJ, Moddel C, Alison JA, McKenzie DK, McKeough ZJ. Home-based telerehabilitation via real-time videoconferencing improves endurance exercise capacity in patients with COPD: The randomized controlled TeleR Study. Respirology. 2017;22(4):699-707.
27. Vianello A, Fusello M, Gubian L, Rinaldo C, Dario C, Concas A, et al. Home telemonitoring for patients with acute exacerbation of chronic obstructive pulmonary disease: a randomized controlled trial. BMC pulmonary medicine. 2016;16(1):157.
