# Supplementary material for: Chronic Obstructive Pulmonary Disease Patients’ Acceptance in E-Health Clinical Trials
Source: Int J Environ Res Public Health. 2021 May 14;18(10):5230. doi: 10.3390/ijerph18105230 (PMC8156037; doi:10.3390/ijerph18105230)
Supplement: Supplementary file 1 [file ijerph-18-05230-s001.zip › Supplementary File S4.pdf]

Multimedia Appendix 4. Detailed description of TH interventions and parallel groups.

Intervention	Summary of interventions attached with TH	Control groups
Usual care with self-management education [1-7]	Regular clinic visits for follow-ups or home care visits for follow- ups with individualized self-management education provide using specific mobile apps (i.e., EDGE), living well with COPD website, booklet or face to face education.	Receiving the self-management materials (apps, booklet, and website) without TM.
Tele-rehabilitation program [8-17]	Designed rehabilitation program packages to deliver at the patient home or the community centre. Health professional monitor, the patients, live or via video conference.	Designed rehabilitation program packages to deliver at the patient home or the community centre.
Home care [9, 14, 18, 19]	Patients receive regular home care included health professional's visits for follow-ups, education sessions, and treatment.	Patient receiving the same without TM between visits.
Virtual hospital [20, 21]	Patient transferred to home within 48 hours of admission to be remotely monitored by healthcare practitioners.	Regular hospitalization.

Reference

1. Antoniades NC, Rochford PD, Pretto JJ, Pierce RJ, Gogler J, Steinkrug J, et al. Pilot study of remote telemonitoring in COPD. TELEMEDICINE and e-HEALTH. 2012;18(8):634-40.
2. Berkhof FF, van den Berg JW, Uil SM, Kerstjens HA. Telemedicine, the effect of nurse-initiated telephone follow up, on health status and health-care utilization in COPD patients: A randomized trial. Respirology. 2015;20(2):279-85.
3. Calvo GS, Gómez-Suárez C, Soriano J, Zamora E, Gónzalez-Gamarra A, González-Béjar M, et al. A home telehealth program for patients with severe COPD: the PROMETE study. Respiratory medicine. 2014;108(3):453-62.
4. Cameron-Tucker HL, Wood-Baker R, Joseph L, Walters JA, Schüz N, Walters EH. A randomized controlled trial of telephone-mentoring with home-based walking preceding rehabilitation in COPD. International journal of chronic obstructive pulmonary disease. 2016;11:1991.

5. Chau JP-C, Lee DT-F, Yu DS-F, Chow AY-M, Yu W-C, Chair S-Y, et al. A feasibility study to investigate the acceptability and potential effectiveness of a telecare service for older people with chronic obstructive pulmonary disease. International journal of medical informatics. 2012;81(10):674-82.
6. De San Miguel K, Smith J, Lewin G. Telehealth remote monitoring for community-dwelling older adults with chronic obstructive pulmonary disease. Telemedicine and e-Health. 2013;19(9):652-7.
7. Dinesen B, Haesum LK, Soerensen N, Nielsen C, Grann O, Hejlesen O, et al. Using preventive home monitoring to reduce hospital admission rates and reduce costs: a case study of telehealth among chronic obstructive pulmonary disease patients. Journal of telemedicine and telecare. 2012;18(4):221-5.
8. Farmer A, Williams V, Velardo C, Shah SA, Yu L-M, Rutter H, et al. Self-management support using a digital health system compared with usual care for chronic obstructive pulmonary disease: randomized controlled trial. Journal of medical Internet research. 2017;19(5):e144.
9. Halpin DM, Laing-Morton T, Spedding S, Levy ML, Coyle P, Lewis J, et al. A randomised controlled trial of the effect of automated interactive calling combined with a health risk forecast on frequency and severity of exacerbations of COPD assessed clinically and using EXACT PRO. Primary Care Respiratory Journal. 2011;20(3):324-31.
10. Ho T-W, Huang C-T, Chiu H-C, Ruan S-Y, Tsai Y-J, Yu C-J, et al. Effectiveness of telemonitoring in patients with chronic obstructive pulmonary disease in Taiwan-A randomized controlled trial. Scientific reports. 2016;6:23797.
11. Koff P, Jones RH, Cashman JM, Voelkel NF, Vandivier R. Proactive integrated care improves quality of life in patients with COPD. European Respiratory Journal. 2009;33(5):1031-8.
12. Lewis KE, Annandale JA, Warm DL, Rees SE, Hurlin C, Blyth H, et al. Does home Telemonitoring after pulmonary rehabilitation reduce healthcare use in optimized COPD?? A pilot randomized trial. COPD: Journal of Chronic Obstructive Pulmonary Disease. 2011;7(1):44-50.
13. Lilholt PH, Udsen FW, Ehlers L, Hejlesen OK. Telehealthcare for patients suffering from chronic obstructive pulmonary disease: effects on health-related quality of life: results from the Danish 'TeleCare North'cluster-randomised trial. BMJ open. 2017;7(5):e014587.
14. Pedone C, Chiurco D, Scarlata S, Incalzi RA. Efficacy of multiparametric telemonitoring on respiratory outcomes in elderly people with COPD: a randomized controlled trial. BMC health services research. 2013;13(1):82.
15. Ringbaek TJ, Lavesen M, Lange P. Tablet computers to support outpatient pulmonary rehabilitation in patients with COPD. European clinical respiratory journal. 2016;3(1):31016.
16. Stickland MK, Jourdain T, Wong EY, Rodgers WM, Jendzjowsky NG, MacDonald GF. Using Telehealth technology to deliver pulmonary rehabilitation to patients with chronic obstructive pulmonary disease. Canadian respiratory journal. 2011;18(4):216-20.
17. Tsai LLY, McNamara RJ, Moddel C, Alison JA, McKenzie DK, McKeough ZJ. Home-based telerehabilitation via real-time videoconferencing improves endurance exercise capacity in patients with COPD: The randomized controlled TeleR Study. Respirology. 2017;22(4):699-707.
18. McDowell JE, McClean S, FitzGibbon F, Tate S. A randomised clinical trial of the effectiveness of home-based health care with telemonitoring in patients with COPD. Journal of telemedicine and telecare. 2015;21(2):80-7.
19. Nield M, Hoo GWS. Real-time telehealth for COPD self-management using Skype™. COPD: Journal of Chronic Obstructive Pulmonary Disease. 2012;9(6):611-9.
20. Jakobsen AS, Laursen LC, Rydahl-Hansen S, Østergaard B, Gerds TA, Emme C, et al. Home-based telehealth hospitalization for exacerbation of chronic obstructive pulmonary disease: findings from "the virtual hospital" trial. Telemedicine and E-health. 2015;21(5):364-73.
21. Schou L, Østergaard B, Rydahl-Hansen S, Rasmussen LS, Emme C, Jakobsen AS, et al. A randomised trial of telemedicine-based treatment versus conventional hospitalisation in patients

with severe COPD and exacerbation–effect on self-reported outcome. Journal of telemedicine and telecare. 2013;19(3):160-5.
