# Supplementary material for: Chronic Obstructive Pulmonary Disease Patients’ Acceptance in E-Health Clinical Trials
Source: Int J Environ Res Public Health. 2021 May 14;18(10):5230. doi: 10.3390/ijerph18105230 (PMC8156037; doi:10.3390/ijerph18105230)
Supplement: Supplementary file 1 [file ijerph-18-05230-s001.zip › Supplementary File S5.pdf]

Multimedia Appendix 5. TH Intervention characteristics of included studies.

Authors	Settings	Intervention with TH	Mode of delivery	Frequency and duration	Control group treatment
Antoniades et al., 2012 [1]	Patient home	Online COPD Self-management education	Web-based	1×/day for 12 months	Usual care
Berkhof et al., 2015 [2]	Medical centre & patient home	Phone calls education and follow ups	Telephone	Call / 2 weeks for 6 months	Usual care
Calvo et al, 2014 [3]	Patient home	Home care with TH	Web-based and telephone	1×/day for 7 months	Usual care
Cameron-Tucker et al.,2016 [4]	Medical centre & patient home	Written home exercise with TH	Telephone	Call / 2 weeks for 2 weeks	Usual care
Chau et al., 2012 [5]	Patient home	Telecare services	Web-base and telephone	3×/day for 2 menthes	Community services
De San Miguel et al, 2013 [6]	Patient home	Written COPD Self-management education	Telephone	1×/day for 6 months	Usual care
Dinesen et al 2012 [7]	Patient home	Telerehabilitation	Video calls	1×/day for 4 months	Instructional book
Farmer et al., 2017 [8]	Patient home	COPD self-management education with TH	Web-based	1×/day for 12 months	COPD self-management education without TH
Halpin et al., 2011 [9]	Patient home	Automated text messages system	Text message	1×/day for 4 months	NR
Ho et al., 2016 [10]	Patient home	Telemonitoring	Web-based and telephone	1×/day for 2 months	Usual care
Jakobsen et al., 2015 [11]	Patient home	Virtual hospital	NR	1×/day for 6 months	Regular hospitalization
Koff et al., 2009 [12]	Patient home	Online COPD Self-management education	Web-base and telephone	1×/day for 3 months	Usual care
Lewis et al., 2011 [13]	Patient home	Home monitoring	telephone	2×/day for 6 months	Usual care
Lilholt et al., 2017 [14]	Patient home & community centre	Telerehabilitation	Web-based, and telephone	NR	Usual rehabilitation
McDowell ,2015 [15]	Patient home	Home-based health care	Telephone	1×/day for 6 months	Usual care
Nield et al., 2012 [16]	Patient home	Online COPD Self-management education	Video calls	1×/week for 1 month	Usual care and face -to-face education
Pedone et al., 2013 [17]	Patient home	Telemonitoring	Web-based, telephone, and algorithm	1× /day for 9 months	Usual care
Pinnock et al., 2013 [18]	Patient home	Online COPD Self-management education	Web-based, telephone, and algorithm	1× /day for 12 months	Usual care
Ringbeak et al., 2015 [19]	Patient home	Online COPD Self-management education and home exercise	Video calls	3× /week for 6 months	Usual care
Ringbeak etal.,2016 [20]	Patient home& outpatient	Online COPD Self-management education and home exercise	Web-based, video calls	NR	Usual PR
Schou et al., 2013 [21]	Patient home	NR	Video calls	1× /day for 3 months	Regular hospitalization.
Shany et al ,2017 [22]	Patient home	Online COPD, Self-management education	Web-based and telephone	1× /day for 12 months	Standard home care

Stickland et al., 2011 [23]	Community centre	Online COPD Self-management education	Web-based and video calls	2×/ week	Usual PR
Tabak et al., 2014 [24]	Patient home	Online COPD Self-management education	Web-based and Video calls	1× /day for 9 months	Usual care
Trappenburg et al.,2008 [25]	Patient home	Software COPD Self-management education	Telephone	1× /day for 6 months	Usual care
Tsai et al.,2017 [26]	Patient home	Online COPD Self-management education and home exercise	Video calls	3×/ week	Usual PR
Vianello et al., 2016 [27]	Hospital	Online COPD Self-management education	Web-based and telephone	1× /day for 12 months	Usual care

Abbreviations: N: Number; ×: Time (s); /: every; PR: Pulmonary rehabilitations; NR: Not reported.

References

1. Antoniades NC, Rochford PD, Pretto JJ, Pierce RJ, Gogler J, Steinkrug J, et al. Pilot study of remote telemonitoring in COPD. TELEMEDICINE and e-HEALTH. 2012;18(8):634-40.
2. Berkhof FF, van den Berg JW, Uil SM, Kerstjens HA. Telemedicine, the effect of nurse-initiated telephone follow up, on health status and health-care utilization in COPD patients: A randomized trial. Respirology. 2015;20(2):279-85.
3. Calvo GS, Gómez-Suárez C, Soriano J, Zamora E, Gónzalez-Gamarra A, González-Béjar M, et al. A home telehealth program for patients with severe COPD: the PROMETE study. Respiratory medicine. 2014;108(3):453-62.
4. Cameron-Tucker HL, Wood-Baker R, Joseph L, Walters JA, Schüz N, Walters EH. A randomized controlled trial of telephone-mentoring with home-based walking preceding rehabilitation in COPD. International journal of chronic obstructive pulmonary disease. 2016;11:1991.
5. Chau JP-C, Lee DT-F, Yu DS-F, Chow AY-M, Yu W-C, Chair S-Y, et al. A feasibility study to investigate the acceptability and potential effectiveness of a telecare service for older people with chronic obstructive pulmonary disease. International journal of medical informatics. 2012;81(10):674-82.
6. De San Miguel K, Smith J, Lewin G. Telehealth remote monitoring for community-dwelling older adults with chronic obstructive pulmonary disease. Telemedicine and e-Health. 2013;19(9):652-7.
7. Dinesen B, Haesum LK, Soerensen N, Nielsen C, Grann O, Hejlesen O, et al. Using preventive home monitoring to reduce hospital admission rates and reduce costs: a case study of telehealth among chronic obstructive pulmonary disease patients. Journal of telemedicine and telecare. 2012;18(4):221-5.
8. Farmer A, Williams V, Velardo C, Shah SA, Yu L-M, Rutter H, et al. Self-management support using a digital health system compared with usual care for chronic obstructive pulmonary disease: randomized controlled trial. Journal of medical Internet research. 2017;19(5):e144.
9. Halpin DM, Laing-Morton T, Spedding S, Levy ML, Coyle P, Lewis J, et al. A randomised controlled trial of the effect of automated interactive calling combined with a health risk forecast on frequency and severity of exacerbations of COPD assessed clinically and using EXACT PRO. Primary Care Respiratory Journal. 2011;20(3):324-31.
10. Ho T-W, Huang C-T, Chiu H-C, Ruan S-Y, Tsai Y-J, Yu C-J, et al. Effectiveness of telemonitoring in patients with chronic obstructive pulmonary disease in Taiwan-A randomized controlled trial. Scientific reports. 2016;6:23797.
11. Jakobsen AS, Laursen LC, Rydahl-Hansen S, Østergaard B, Gerds TA, Emme C, et al. Home-based telehealth hospitalization for exacerbation of chronic obstructive pulmonary disease: findings from "the virtual hospital" trial. Telemedicine and E-health. 2015;21(5):364-73.

12. Koff P, Jones RH, Cashman JM, Voelkel NF, Vandivier R. Proactive integrated care improves quality of life in patients with COPD. European Respiratory Journal. 2009;33(5):1031-8.
13. Lewis KE, Annandale JA, Warm DL, Rees SE, Hurlin C, Blyth H, et al. Does home Telemonitoring after pulmonary rehabilitation reduce healthcare use in optimized COPD?? A pilot randomized trial. COPD: Journal of Chronic Obstructive Pulmonary Disease. 2011;7(1):44-50.
14. Lilholt PH, Udsen FW, Ehlers L, Hejlesen OK. Telehealthcare for patients suffering from chronic obstructive pulmonary disease: effects on health-related quality of life: results from the Danish 'TeleCare North'cluster-randomised trial. BMJ open. 2017;7(5):e014587.
15. McDowell JE, McClean S, FitzGibbon F, Tate S. A randomised clinical trial of the effectiveness of home-based health care with telemonitoring in patients with COPD. Journal of telemedicine and telecare. 2015;21(2):80-7.
16. Nield M, Hoo GWS. Real-time telehealth for COPD self-management using Skype™. COPD: Journal of Chronic Obstructive Pulmonary Disease. 2012;9(6):611-9.
17. Pedone C, Chiurco D, Scarlata S, Incalzi RA. Efficacy of multiparametric telemonitoring on respiratory outcomes in elderly people with COPD: a randomized controlled trial. BMC health services research. 2013;13(1):82.
18. Pinnock H, Hanley J, McCloughan L, Todd A, Krishan A, Lewis S, et al. Effectiveness of telemonitoring integrated into existing clinical services on hospital admission for exacerbation of chronic obstructive pulmonary disease: researcher blind, multicentre, randomised controlled trial. Bmj. 2013;347:f6070.
19. Ringbæk T, Green A, Laursen LC, Frausing E, Brøndum E, Ulrik CS. Effect of tele health care on exacerbations and hospital admissions in patients with chronic obstructive pulmonary disease: a randomized clinical trial. International journal of chronic obstructive pulmonary disease. 2015;10:1801.
20. Ringbaek TJ, Lavesen M, Lange P. Tablet computers to support outpatient pulmonary rehabilitation in patients with COPD. European clinical respiratory journal. 2016;3(1):31016.
21. Schou L, Østergaard B, Rydahl-Hansen S, Rasmussen LS, Emme C, Jakobsen AS, et al. A randomised trial of telemedicine-based treatment versus conventional hospitalisation in patients with severe COPD and exacerbation–effect on self-reported outcome. Journal of telemedicine and telecare. 2013;19(3):160-5.
22. Shany T, Hession M, Pryce D, Roberts M, Basilakis J, Redmond S, et al. A small-scale randomised controlled trial of home telemonitoring in patients with severe chronic obstructive pulmonary disease. Journal of telemedicine and telecare. 2017;23(7):650-6.
23. Stickland MK, Jourdain T, Wong EY, Rodgers WM, Jendzjowsky NG, MacDonald GF. Using Telehealth technology to deliver pulmonary rehabilitation to patients with chronic obstructive pulmonary disease. Canadian respiratory journal. 2011;18(4):216-20.
24. Tabak M, Brusse-Keizer M, van der Valk P, Hermens H, Vollenbroek-Hutten M. A telehealth program for self-management of COPD exacerbations and promotion of an active lifestyle: a pilot randomized controlled trial. International journal of chronic obstructive pulmonary disease. 2014;9:935.
25. Trappenburg JC, Niesink A, de Weert-van Oene GH, van der Zeijden H, van Snippenburg R, Peters A, et al. Effects of telemonitoring in patients with chronic obstructive pulmonary disease. Telemedicine and e-Health. 2008;14(2):138-46.
26. Tsai LLY, McNamara RJ, Moddel C, Alison JA, McKenzie DK, McKeough ZJ. Home-based telerehabilitation via real-time videoconferencing improves endurance exercise capacity in patients with COPD: The randomized controlled TeleR Study. Respirology. 2017;22(4):699-707.
27. Vianello A, Fusello M, Gubian L, Rinaldo C, Dario C, Concas A, et al. Home telemonitoring for patients with acute exacerbation of chronic obstructive pulmonary disease: a randomized controlled trial. BMC pulmonary medicine. 2016;16(1):157.
