# Supplementary figures and images for: Chronic Obstructive Pulmonary Disease Patients’ Acceptance in E-Health Clinical Trials
Source: Int J Environ Res Public Health. 2021 May 14;18(10):5230. doi: 10.3390/ijerph18105230 (PMC8156037; doi:10.3390/ijerph18105230)

## Multimedia Appendix 6. Forest plot of acceptance rate in TH intervention.

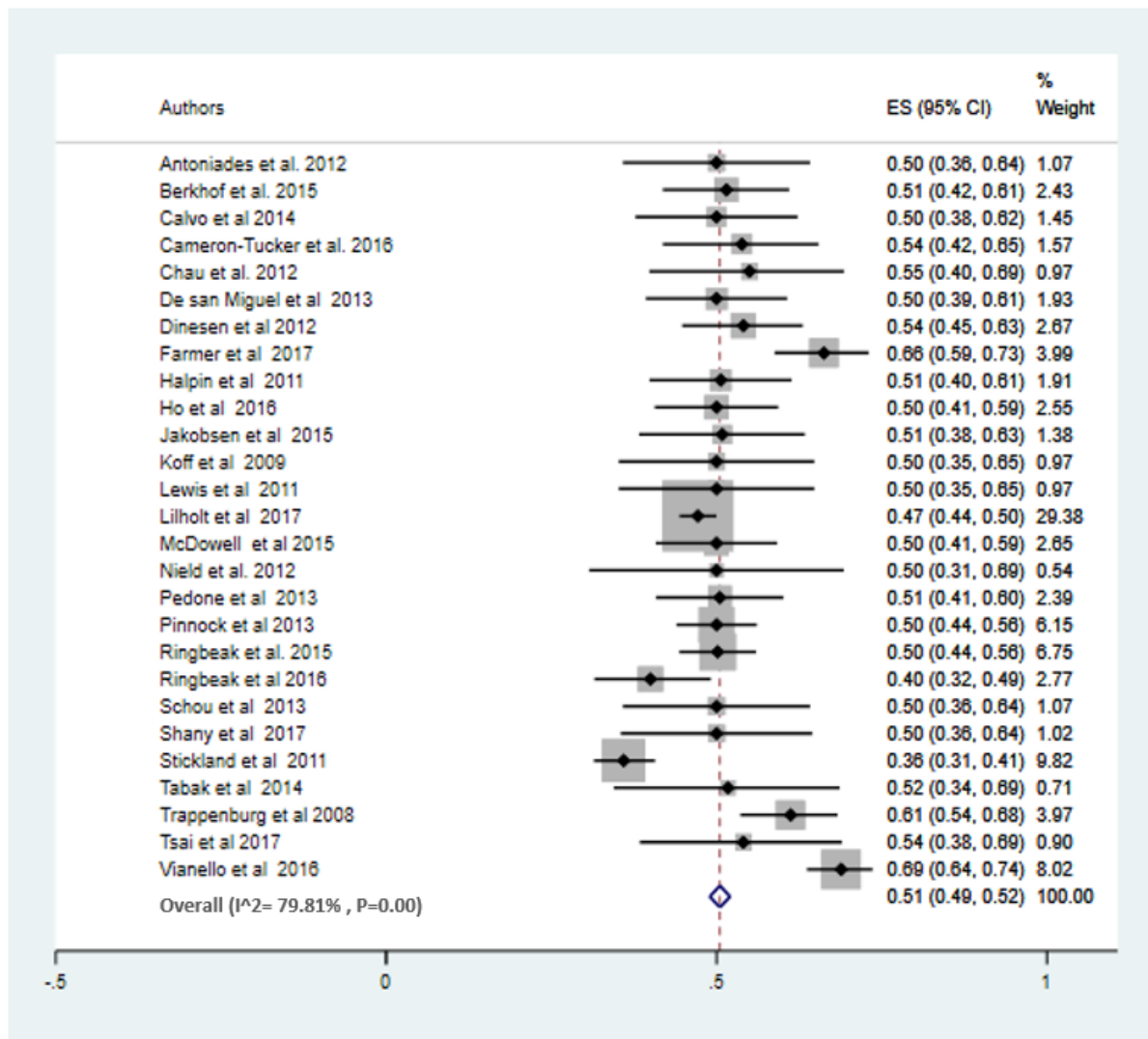

Supplement: Supplementary file 1 [file ijerph-18-05230-s001.zip › Supplementary File S6.pdf]

## Multimedia Appendix 7: Forest plot of dropout rate in TH intervention.

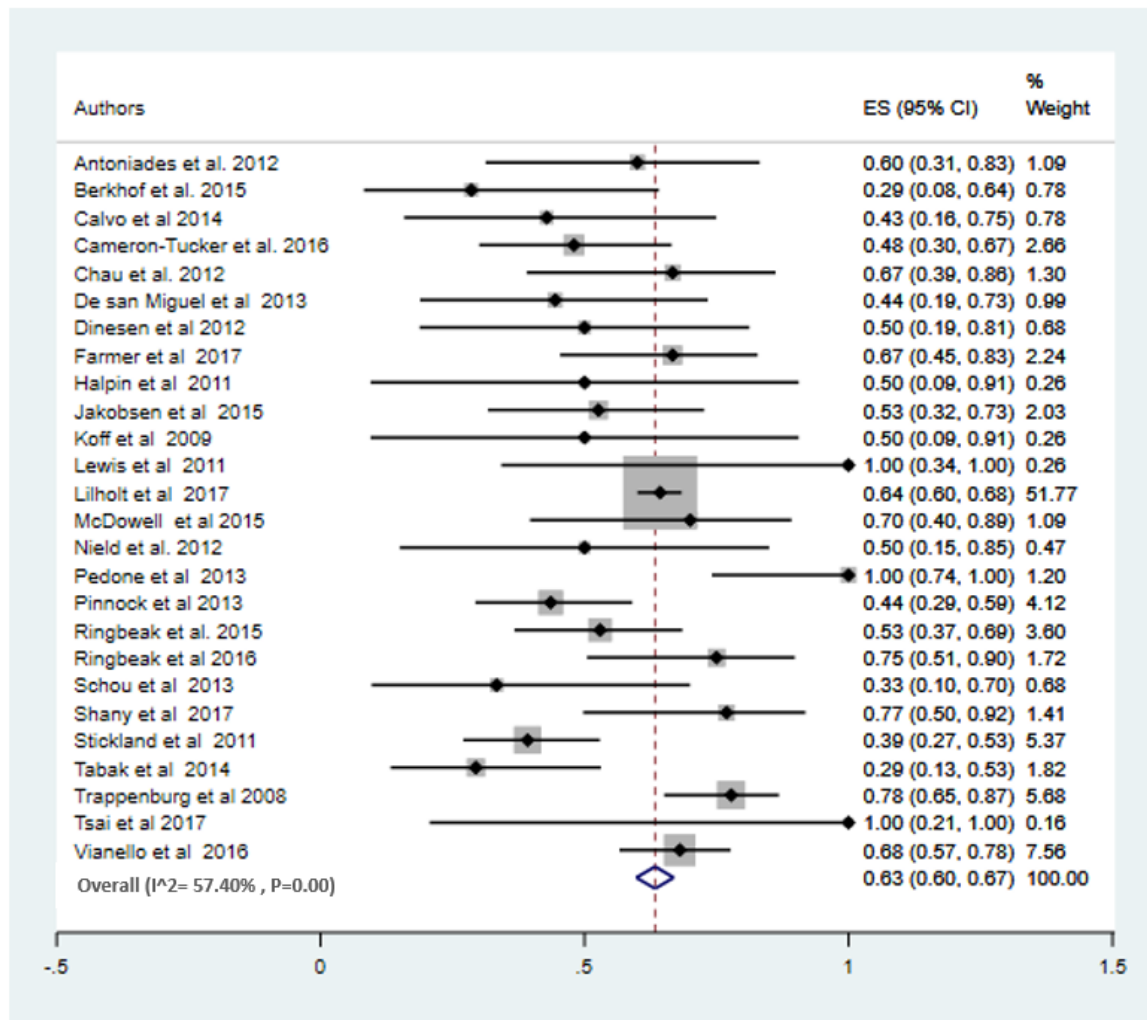

Supplement: Supplementary file 1 [file ijerph-18-05230-s001.zip › Supplementary File S7.pdf]

## Multimedia Appendix 8. Forest plot of acceptance rate in controls

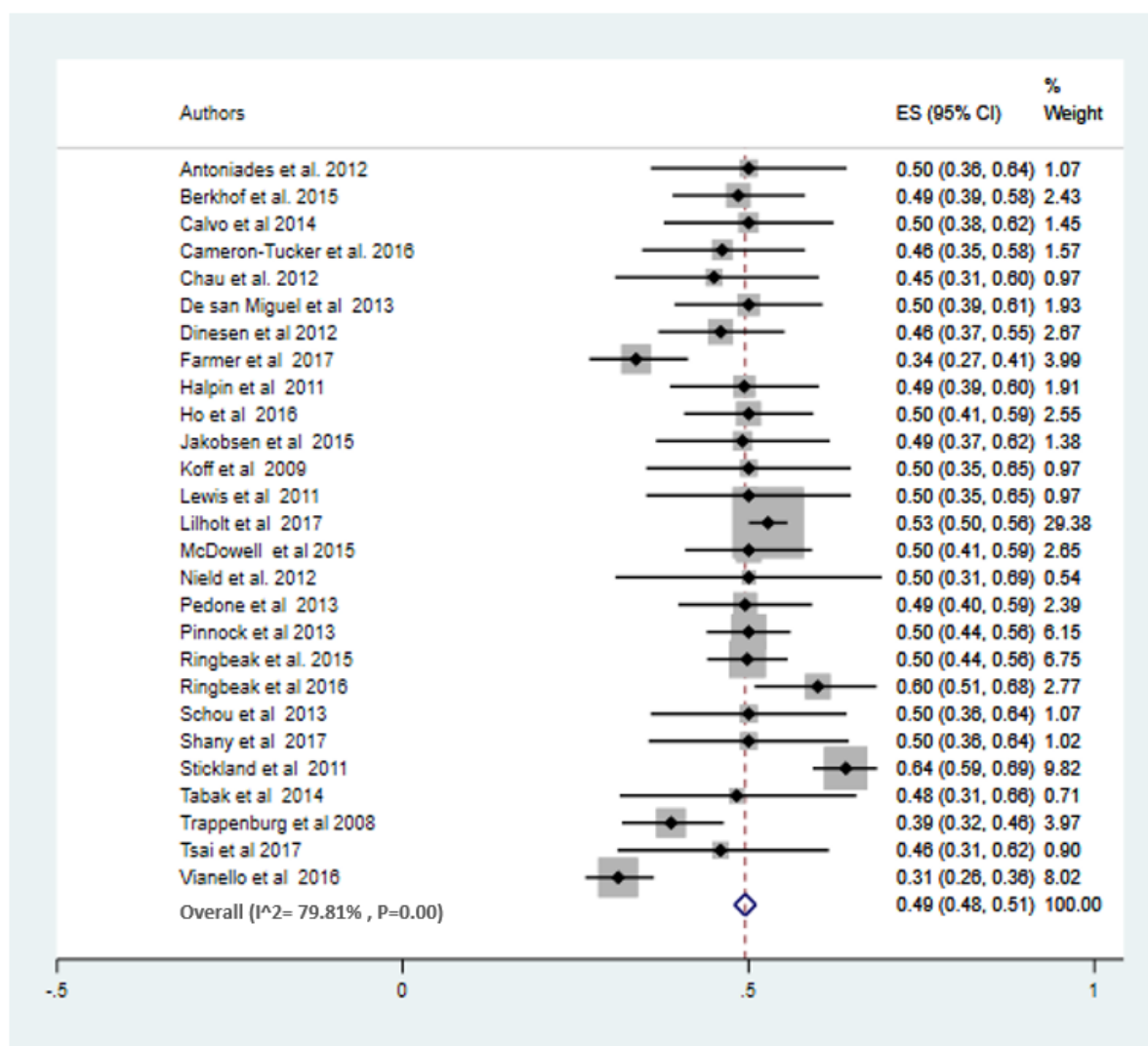

Supplement: Supplementary file 1 [file ijerph-18-05230-s001.zip › Supplementary File S8.pdf]

## Multimedia Appendix 9. Forest plot of dropout rat in controls.

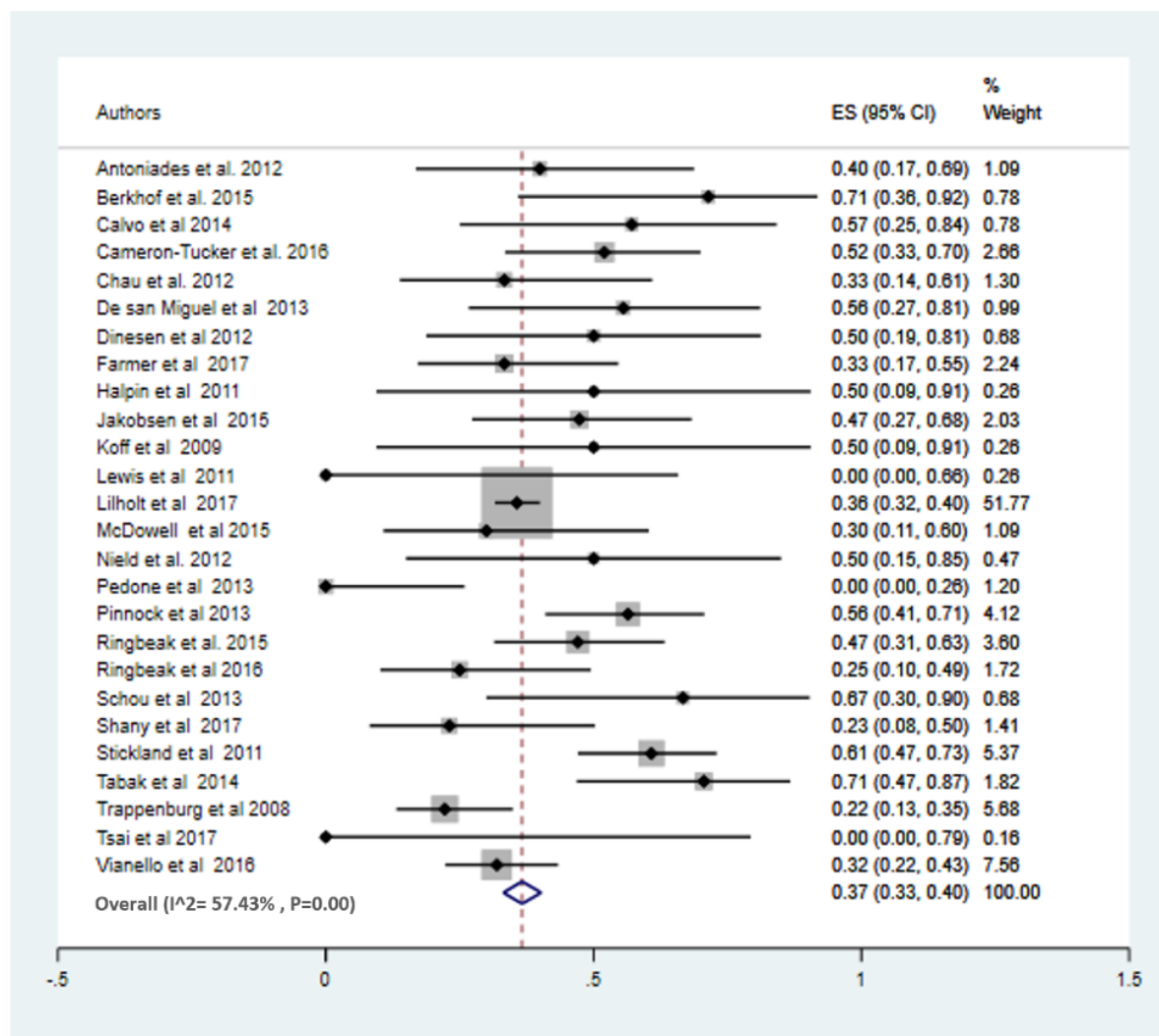

Supplement: Supplementary file 1 [file ijerph-18-05230-s001.zip › Supplementary File S9.pdf]
